# Origin, course, and distribution of the posterior femoral cutaneous nerve and the spatial relationship among its branches

**DOI:** 10.1007/s12565-023-00721-x

**Published:** 2023-04-05

**Authors:** Areeya Jiamjunyasiri, Masahiro Tsutsumi, Satoru Muro, Keiichi Akita

**Affiliations:** 1 Department of Clinical Anatomy, Graduate School of Medical and Dental Sciences, Tokyo Medical and Dental University (TMDU), Yushima 1-5-45, Bunkyo-ku, Tokyo 113-8519 Japan; 2 Inclusive Medical Sciences Research Institute, Morinomiya University of Medical Sciences, Osaka, Japan

**Keywords:** Posterior femoral cutaneous nerve, Perforating cutaneous nerve, Sacrotuberous ligament, Inferior cluneal nerves, Perineal branches

## Abstract

This study aimed to elucidate the origin, course, and distribution of the branches of the posterior femoral cutaneous nerve, considering the segmental and dorsoventral compositions of the sacral plexus, including the pudendal nerve. The buttocks and thighs of five cadavers were analyzed bilaterally. The branches emerged from the sacral plexus, which was divided dorsally to ventrally into the superior gluteal, inferior gluteal, common peroneal, tibial, and pudendal nerves. It descended lateral to the ischial tuberosity and comprised the thigh, gluteal, and perineal branches. As for the thigh and gluteal branches, the dorsoventral order of those originating from the sacral plexus corresponded to the lateromedial order of their distribution. However, the dorsoventral boundary was displaced at the inferior margin of the gluteus maximus between the thigh and gluteal branches. The perineal branch originated from the ventral branch of the nerve roots. In addition, the pudendal nerve branches, which ran medially to the ischial tuberosity, were distributed in the medial part of the inferior gluteal region. These branches should be distinguished from the gluteal branches; the former should be classified as the medial inferior cluneal nerves and the latter as the lateral ones. Finally, the medial part of the inferior gluteal region was distributed by branches of the dorsal sacral rami, which may correspond to the medial cluneal nerves. Thus, the composition of the posterior femoral cutaneous nerve is considered necessary when considering the dorsoventral relationships of the sacral plexus and boundaries of the dorsal and ventral rami.

## Introduction

The posterior femoral cutaneous nerve (PFC), as suggested by its name N. cutaneus femoris posticus communis (the common posterior femoral cutaneous nerve), given by Eisler (1891), has one of the most complex cutaneous branches that are not only distributed in the posterior thighs but also in the gluteal and perineal regions (Romanes 1972; Standring 2015). The PFC runs lateral to the ischial tuberosity along the sciatic nerve, descends to the lower border of the gluteus maximus, and gives off three branches. The branches from the PFC are classified into thigh, gluteal, and perineal branches. The thigh branches pass downward from the posterior surface of the thigh deep to the fascia lata. Subsequently, they are distributed to the skin and fascia of the posterior surfaces of the thigh and proximal leg. The gluteal branches are distributed in the inferior half of the gluteal region. In addition, perineal branches are distributed in the lateral part of the perineum. Since the PFC is susceptible to neuropathy (Arnoldussen and Korten 1980; Tong and Haig 2000; Gomceli et al. 2005), which is accompanied by pain (Dellon 2015; Joshi et al. 2017), and can be utilized for nerve block as a therapeutic intervention (Kasper et al. 2014), exploring the complex anatomy of the PFC may be of clinical significance.

In general, most branches of the PFC emerge from the sacral plexus (Romanes 1972; Standring 2015). However, Nakanishi et al. (1976) reported that some branches and roots of the PFC originate from the pudendal nerve and its roots, and regarded the PFC as a nerve originating from both the sacral and pudendal plexuses. Since most studies reported that the sacral plexus could be distinguished from the pudendal plexus (Eisler 1891, 1892; Bardeen and Elting 1991a, b; Nakanishi 1967a, b; Takahashi 1980; Sato 1980), the PFC is complex not only in its distribution but also in its origin. On the contrary, some comparative anatomical studies have reported the caudoventral location of the pudendal nerve (or a nerve homologous to the pudendal nerve) within the sacral plexus (Akita et al. 1992b, 1995; Akita and Yamamoto 1995). Although the pudendal nerve constitutes most of the pudendal plexus, and the segments of the roots of the sacral plexus and pudendal nerve overlap, the dorsoventral relationship between the two plexuses has rarely been described. Therefore, we hypothesized that the complex anatomy of the PFC could be reorganized by focusing on the dorsoventral relationship between the sacral plexus and the pudendal nerve.

In the present study, we examined the segmental and dorsoventral compositions of the nerves of the sacral plexus, including the pudendal nerve. In addition, we investigated the origin, course, and distribution of the branches of the PFC.

## Materials and methods

Ten buttocks and posterior thighs of five cadavers (two males mean age 88.5 years, range 82–95, and three females mean age 85.3 years; range 77–92), donated to the Department of Anatomy, Tokyo Medical and Dental University, were used in this study. Before their death, all donors declared that their remains would be donated to education and study. Our study complied with the Japanese law, titled The Act on Body Donation for Medical and Dental Education, and international ethical guidelines and laws concerning the use of human cadaveric donors in anatomical research. The study design was approved by the Medical Research Ethics Committee of Tokyo Medical and Dental University (approval no.: #M2021-086). The authors hereby confirm that every effort was made to comply with all local and international ethical guidelines and laws concerning the use of human cadaveric donors in anatomical research.

**Fig. 1:**
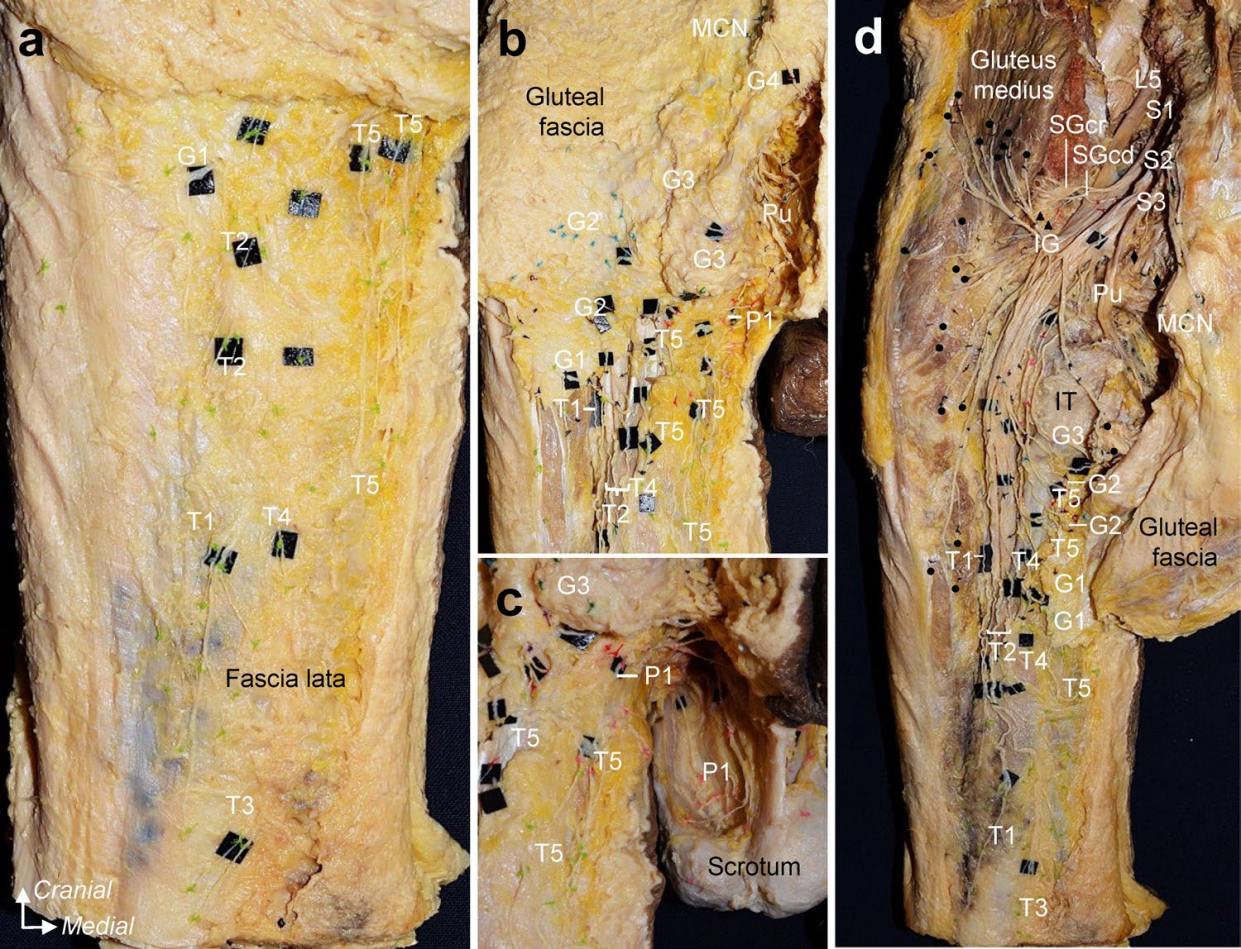
Dissection of the cutaneous branches to the posterior thigh, inferior gluteal, and perineal regions (Specimen no. 3; left, male). **a**: Posterior thigh region. After the removal of the skin, the thigh branches subsequent to penetrating the fascia lata were recorded with colored strings.** b**: Inferior gluteal region. The facia lata was partly cut and removed, preserving the area where the cutaneous nerve penetrates the facia lata as much as possible, and the gluteal branches were recorded with colored strings before removing the gluteal fascia and gluteal maximum. **c**: Perineal region. Perineal branches were also recorded with colored strings. **d**: After recording the point at which each cutaneous nerve penetrated the fascia lata and ran the course concerning the ischial tuberosity and inferior margin of the gluteus maximus, we removed the fascia lata, gluteus maximus, sacrotuberous ligament, and bony elements of the sacrum and ilium to analyze the origin of the cutaneous nerves. T, G, and P indicate the thigh, gluteal, and perineal cutaneous branches, respectively, and are numbered from the lateral side. Circle = nerve to the gluteus maximus; Diamond, nerve to coccygeus and levator ani; IG, inferior gluteal nerve; IT, ischial tuberosity; MCN, medial cranial nerve; Pu, pudendal nerve; SGcd, caudal part of the superior gluteal nerve; SGcr, cranial part of the superior gluteal nerve; Ti, tibial nerve; triangle, nerve to piriformis

All the specimens were fixed in 8% formalin and preserved in 30% ethanol. First, the posterior thigh, inferior gluteal, and perineal skin were removed while recording the distribution of cutaneous branches with colored strings (Fig. 1a–c). Second, the fascia lata of the posterior thigh was removed, and the gluteus maximus insertion was detached and reflected proximally. During this process, we recorded where each cutaneous nerve penetrated the fascia lata and the course of the ischial tubercle and inferior margin of the gluteus maximus. Finally, the sacrotuberous ligament and bony elements of the sacrum and ilium were partly removed to examine the origin of the cutaneous nerves and their relationship to the main nerves comprising the sacral and pudendal plexus, such as the superior and inferior gluteal, common peroneal, tibial, and pudendal nerves (Fig. 1d). All data were recorded as photographs and schematic illustrations and were compared among all specimens.

## Results

**Fig. 2:**
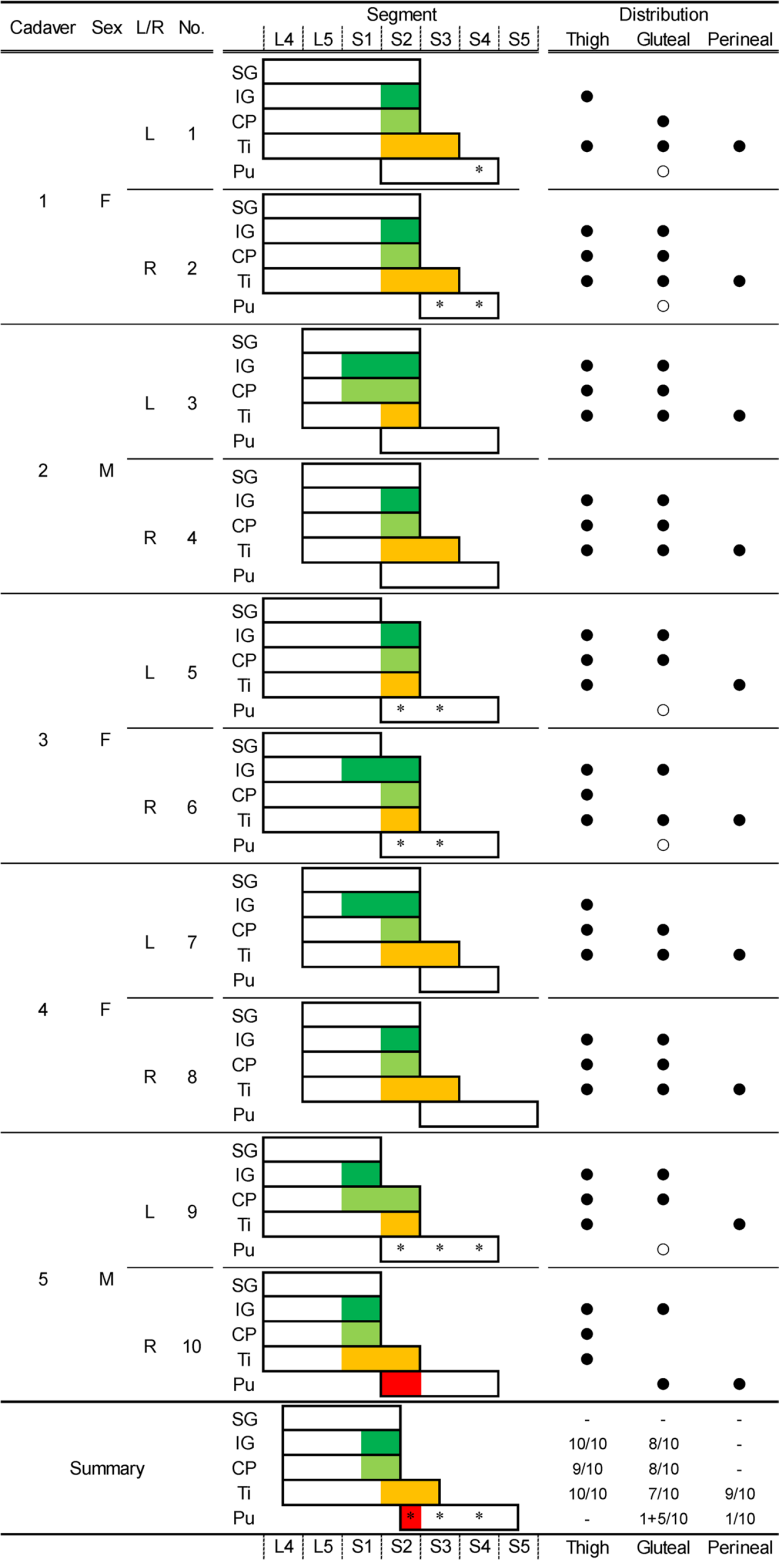
Relationships between the origin and distribution of the posterior femoral cutaneous nerve. The figure shows the segmental arrangement of the main nerves of the sacral and pudendal plexuses and their relation to the posterior femoral cutaneous nerve in all specimens. Colored areas (green, inferior gluteal nerve; light green, common peroneal nerve; orange, tibial nerve; and red, pudendal nerve) indicate the origin and segment of the posterior femoral cutaneous nerve. The black circle indicates the presence of the posterior femoral cutaneous nerve distributed in the thigh, gluteal, and perineal regions. Asterisks and white circles indicate the presence and segment of the cutaneous branches originating from the pudendal nerve to the gluteal region. CP, common peroneal nerve; F, female; IG, inferior gluteal nerve; L/R, left or right sides; M, male; Pu, pudendal nerve; Ti = Tibial nerve.

The PFC comprised branches distributed to the thigh, inferior gluteal, and perineal regions. The branches of the PFC originated from the main nerves and/or the roots of the main nerves of the sacral plexus, such as the inferior gluteal, common peroneal, and tibial nerves, in all specimens. The main nerves of the sacral plexus were arranged dorsally to ventrally in the superior gluteal, inferior gluteal, common peroneal, tibial, and pudendal nerves in all specimens. The origins of the PFC branches distributing to the thigh, inferior gluteal, and perineal regions of the sacral plexus are shown in Fig. 2.

**Fig. 3:**
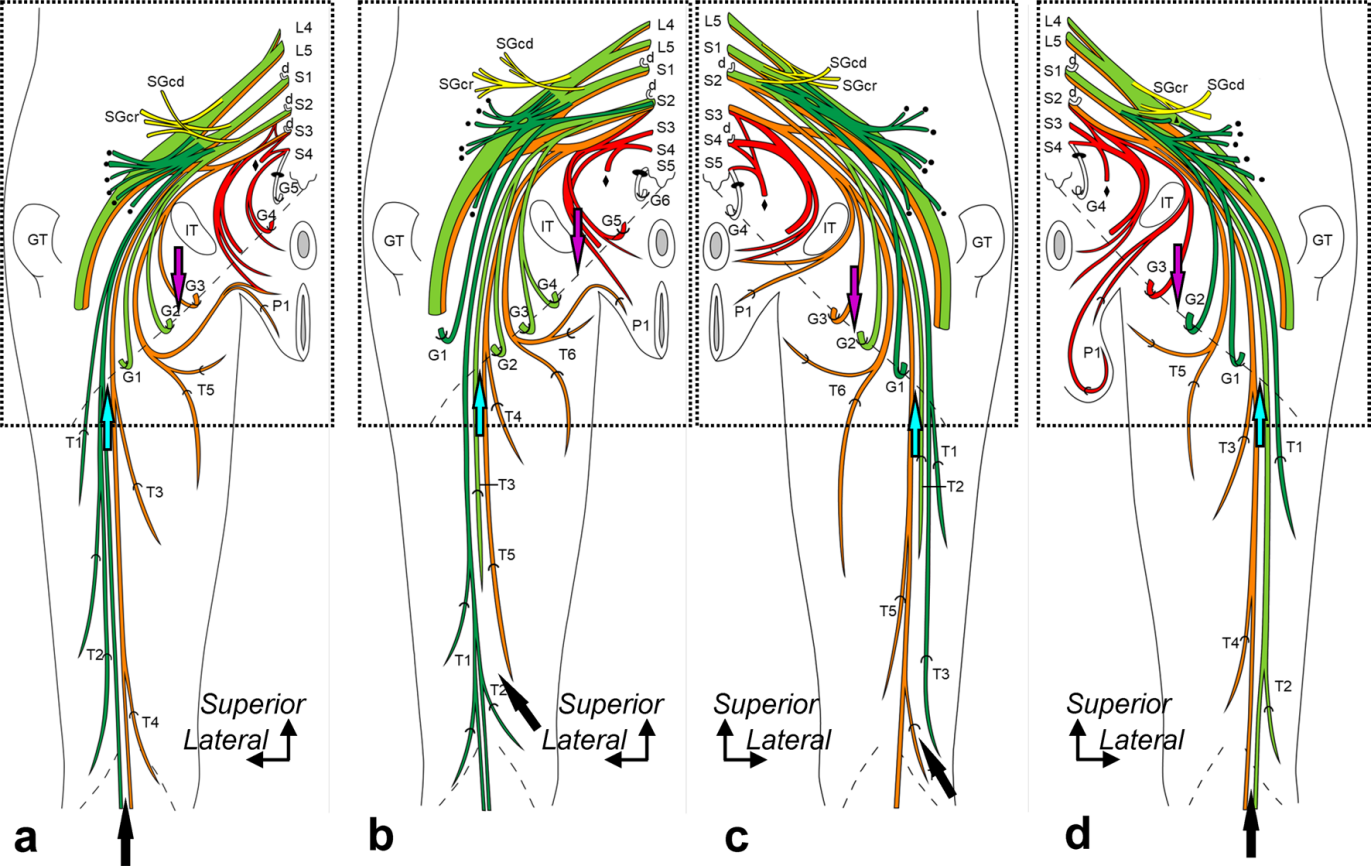
Origin, course, and distribution of the posterior femoral cutaneous nerve. Schematic illustrations of the buttocks and posterior thighs. **a**: Specimen No. 1 (left, female), **b**: Specimen No. 5 (left, female), **c**: Specimen No. 8 (right, female), and **d**: Specimen No. 10 (right, male). The thigh, gluteal, and perineal cutaneous branches are indicated by T, G, and P, respectively, numbered from the lateral side. The dotted oblique line indicates the inferior margin of the gluteus maximus, and the recurrent course of the distal cutaneous branches is shown in Fig. 4 (indicated by the boxed region). The posterior femoral cutaneous nerve originates from the following nerves comprising the sacral and pudendal plexus: inferior gluteal (green), common peroneal (light green), tibial (orange), and pudendal nerves (red). Based on the stratification relationship among these nerves, the blue and purple arrows at the inferior margin of the gluteus maximus indicate the dorsoventral boundaries of the thigh and gluteal cutaneous branches, respectively. The black arrow at the distal posterior thigh indicates the dorsoventral boundary of the thigh. Circle, nerve to the gluteus maximus; d, dorsal rami; Diamond, nerve to coccygeus and levator ani; GT, greater trochanter; L, (e.g., L4) the lumbar nerve; IT, ischial tuberosity; S, (e.g., S1) the sacral nerve; SGcd, caudal part of the superior gluteal nerve; SGcr, cranial part of the superior gluteal nerve; and triangle, nerve to the piriformis.

As for the thigh branches of the PFC, those originating from the more dorsal nerves (inferior gluteal nerve, green colored in Fig. 3a–d; common peroneal nerve, light green colored in Fig. 3a–d) were distributed more laterally. In contrast, those originating from the more ventral nerves (tibial nerve, orange colored in Fig. 3a–d) were distributed more medially. As for the gluteal branches of the PFC, those originating from the inferior gluteal, common peroneal, and tibial nerves were radially distributed. One of the gluteal branches originated from the pudendal nerve in one specimen (red-colored, Fig. 3d). Unlike the other gluteal branches, this branch did not form a plexus with the other main nerves of the sacral plexus, showing an independent running course from the other gluteal branches. It was distributed in the most medial region. The perineal branches of the PFC originated from the tibial nerve in 9 specimens and the root of the pudendal nerve in one specimen.

**Fig. 4:**
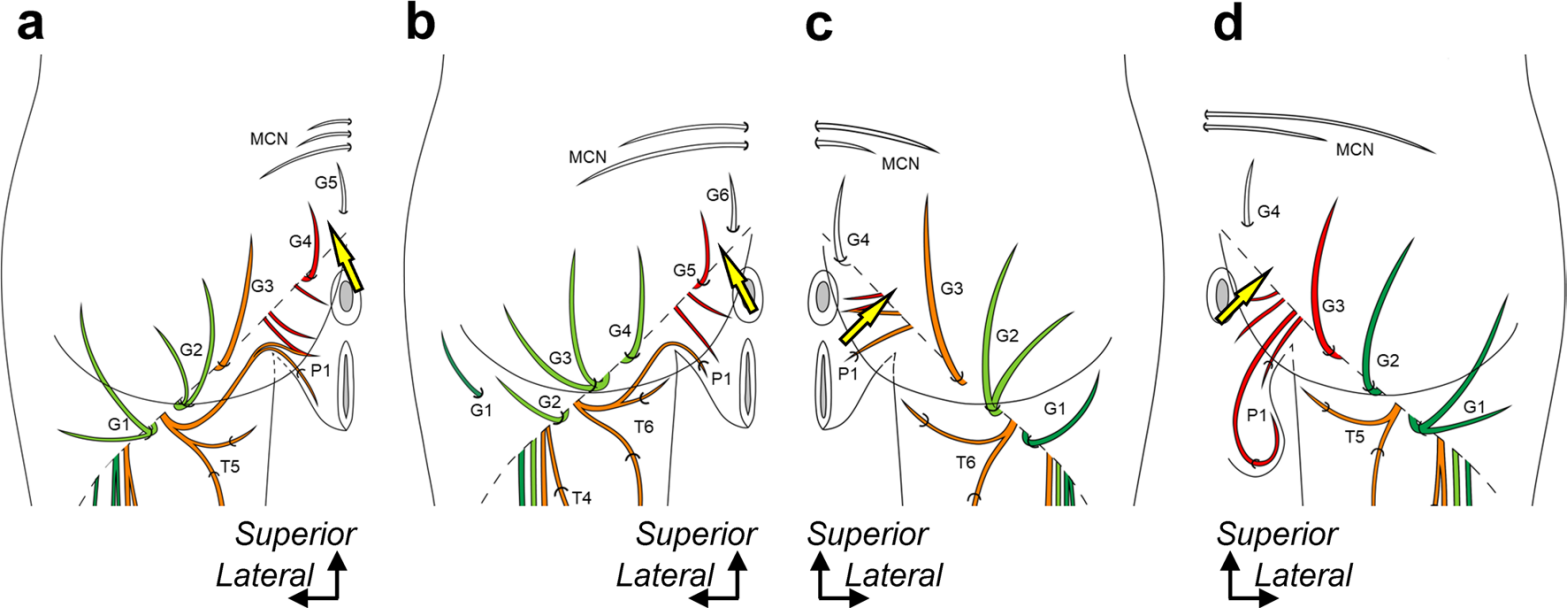
Posterior femoral cutaneous nerve distribution in the gluteal region. Schematic illustrations of the buttocks. **a**: Specimen No. 1 (left, female), **b**: Specimen No. 5 (left, female), **c**: Specimen No. 8 (right, female), and **d**: Specimen No. 10 (right, male). The corresponding illustrations deep to the gluteus maximus of **a**–**d** are shown in the boxed region of Fig. 3a–d. The thigh, gluteal, and perineal cutaneous branches are indicated by T, G, and P, respectively, numbered from the lateral side. The cutaneous branches are colored based on their origin, such as the inferior gluteal (green), common peroneal (light green), tibial (orange), and pudendal nerves (red). The yellow arrow indicates the boundary between the ventral and dorsal rami. Dotted oblique line = inferior margin of the gluteus maximus, and MCN = medial cluneal nerve.

The dorsoventral boundary between the cutaneous branches originating from the dorsal (inferior gluteal and common peroneal nerves) and ventral (tibial and pudendal nerves) nerves of the sacral plexus varied according to the specimens. Regarding the thigh cutaneous branches, although the dorsoventral boundary at the inferior margin of the gluteus maximus was located approximately on the lateral one-third of the thigh in all specimens (Fig. 3a–d; blue arrow), those at the distal posterior thigh varied according to the specimens (Fig. 3a–d; black arrow). In three of ten specimens, the dorsoventral boundary extended to the posterior part of the knee (Fig. 3a, d; black arrow). In the other seven specimens, the posterior part of the knee was occupied only by the dorsal (cutaneous branches originating from the inferior gluteal nerve and/or the common peroneal nerve; six specimens, Fig. 3b) or ventral nerves (those originating from the tibial nerve; one specimen, Fig. 3c). As for the gluteal cutaneous branches, the dorsoventral boundary at the inferior margin of the gluteus maximus was lateral to the ischial tubercle in eight specimens (Fig. 3a, c, d; purple arrow). The other two specimens were located approximately on the ischial tubercle (Fig. 3b; purple arrow). However, the PFC was mainly distributed in the inferior gluteal region, and two types of cutaneous branches running medially to the ischial tubercle were also distributed (Fig. 4). The branches originated from the pudendal nerve and/or the roots of the pudendal nerve (five of ten specimens; asterisk and white circle in Fig. 2). The positions of the dorsoventral boundary between the cutaneous branches originating from the dorsal and ventral nerves of the sacral plexus were displaced from one another in the thigh and gluteal regions (Fig. 3). In addition, a branch pierced the gluteus maximus and was distributed in the lateral part of the inferior gluteal region (Fig. 4b).

Branches from the dorsal rami of the fourth or fifth sacral nerve (S4 or S5) were frequently distributed in the medial region of the inferior gluteal region, and the branches penetrated the proximal region of the sacrotuberous ligament (8/10 specimens) (Fig. 3). In other words, the boundary between the ventral and dorsal rami was observed in the inferior gluteal region of these specimens (Fig. 4; yellow arrows). Furthermore, the cutaneous branches from the dorsal rami also penetrated the inferomedial region of the gluteus maximus in five specimens (Fig. 4a, b, d). They curled around the inferior margin of the gluteus maximus in the other three specimens (Fig. 4c).

## Discussion

The present study revealed that the branches of the PFC originating from the dorsal nerves of the sacral plexus, the common peroneal and inferior gluteal nerves, are distributed laterally in this order in the posterior thigh and inferior gluteal region. In contrast, those from the ventral, tibial, and pudendal nerves are distributed medially in this order in the perineal region. However, the dorsoventral boundary was displaced from one another at the inferior margin of the gluteus maximus between the thigh and the gluteal cutaneous branches. The perineal branch originates from the PFC’s ventral branch of the roots. In addition, the branches from the pudendal nerve and dorsal rami of S4 and S5 were distributed on the medial part of the inferior gluteal region.

### PFC composition based on the dorsoventral relationships between the sacral plexus and pudendal nerve

The PFC is a complex of cutaneous branches originating from the dorsal and ventral divisions of the sacral plexus (Romanes 1972; Standring 2015). The PFC comprises various combinations of branches, and Nakanishi et al. (1976) reported various patterns of their origin. Based on comparative anatomical studies, Nakanishi et al. (1976) considered that the PFC was primarily a division of the pudendal nerve, and the nerve expanded to the inferior limb by the medial rotation of the limb. However, most branches of the PFC originate from the nerves and roots of the sacral plexus, and the branches from the pudendal nerve are only distributed to the small medial regions. Therefore, the main trunk of the PFC may be composed of cutaneous nerves of the lower limb, which may have secondarily shared its distribution area with branches of the pudendal nerve.

In the present study, the main nerves of the sacral plexus were classified dorsoventrally into superior gluteal, inferior gluteal, common peroneal, tibial, and pudendal nerves, as in previous studies (Akita et al. 1992b, 1995; Akita and Yamamoto 1995). The superior gluteal nerve has no cutaneous branches in the inferior gluteal region. However, the cutaneous branches originating from each main nerve of the sacral plexus were distributed laterally and medially. Therefore, the dorsoventral order of the origin of the branches of the PFC from the sacral plexus corresponds to the lateromedial order of their distribution. In addition, the perineal branch was distributed in the superomedial region of the posterior thigh. The perineal branch may then be the medial branch of the thigh branches.

Thus, a correlation between the nerve origin and distribution was evident. According to Figs. 2 and 3, in most cases, the segment of the femoral branches that originates from the dorsal division of the sacral plexus is one segment higher than the segment of the branch that originates from the ventral division because the caudal segment of the common peroneal nerve is mostly one segment higher than the caudal segment of the tibial nerve. Furthermore, the boundary between the branches originating from the dorsal and ventral divisions of the sacral plexus was observed at the midline of the posterior thigh, which should be located at the boundary between the common peroneal and tibial nerves. Therefore, the lateral thigh branches from the dorsal division tend to have higher originating segments, and the medial thigh branches from the ventral division tend to have lower originating segments.

In contrast, the boundary positions between branches originating in the ventral and dorsal divisions of the sacral plexus differed between the gluteal and thigh branches. The gluteal branches originating from the sacral plexus are inverted at the inferior border of the gluteus maximus. The boundary displacement observed in the present study may have been caused by a downward shift of the inferior border of the gluteus maximus with the growth of the muscle, which in turn shifts the courses of the gluteal branches.

### Perineal branch of the PFC

The perineal branch ran along the lateral surface of the ischial tuberosity and turned medially at the ischial tuberosity. It branched into the upper medial part of the thigh and the lateral part of the perineum. Therefore, the branch was distributed in the transitional zone between the thigh and perineum. The perineal branch passes ventrally to supply the skin of the scrotum (labium majus) and the root of the penis (clitoris).

Perineal branches were observed in all specimens in the present study, which sometimes communicated with a branch of the pudendal nerve in the perineum. Tubbs et al. (2009) reported that the branch was absent in 15% of the cases. In addition, the branch arose from the thigh branch in 55% of the cases and the inferior cluneal nerve in 30%. Eisler (1891) reported a rare nerve branch penetrating the sacrotuberous ligament and communicating with the PFC. He also found a branch running between the sacrotuberous and sacrospinous ligaments. These nerves, which Eisler (1891) reported, originate from S3. Therefore, branches running from the medial to the ischial tuberosity can compensate for the perineal branch of the PFC.

### Gluteal branches of the PFC

The gluteal branches run along the inferior border of the gluteus maximus and are distributed in the inferior gluteal region. As previously mentioned, the dorsoventral origin of the gluteal branches from the sacral plexus corresponds to the lateromedial order of their distribution. Two problems regarding cutaneous branches distributed in the inferior gluteal region need to be solved. One is the branch penetrating the gluteus maximus muscle in the lateral part. The other is about which branches in the medial part should be considered gluteal branches of the PFC.

In the present study, as shown in Fig. 4b, a branch originating from the inferior gluteal nerve penetrated the gluteus maximus to be distributed to the lateral part of the inferior gluteal region. Akita et al. (1992a) reported that the branches of the superior gluteal nerve frequently penetrate the tensor fasciae latae and gluteus maximus and are distributed in the area surrounded by the regions distributed by the iliohypogastric, inferior cluneal, superior cluneal, and lateral femoral cutaneous nerves. Therefore, the branch that penetrated the gluteus maximus originating from the inferior gluteal nerve could be similar to the cutaneous branches from the superior gluteal nerve. Therefore, the branch should be better distinguished from the gluteal branches of the PFC.

The gluteal branches mainly originate from the inferior gluteal, common peroneal, and tibial nerves. These branches run laterally to the ischial tuberosity beneath the gluteus maximus and recur at the inferior margin of the muscle. However, branches originating from the pudendal nerve are often found in the medial region of the distribution area of these branches. The branches from the pudendal nerve run medially to the ischial tuberosity, and these branches can be distinguished from those lateral to them. Eisler (1891) reported a rare nerve, N. perforans ligamenti sacrotuberosi, in this region, which runs medial to the ischiatic tuberosity and penetrates the sacroiliac ligament to be distributed to the inferior gluteal region. Florian-Rodriguez et al. (2016) also reported a similar perforating nerve of the sacrotuberous ligament. This nerve can also be distinguished from the lateral branches. Therefore, only the lateral branches can be considered the gluteal branches of the PFC. In addition, the lateral and medial gluteal branches should be classified into the lateral and medial inferior cluneal nerves.

Moreover, in the present study, different nerves were found to be distributed in the region medial to the medial branches originating from the pudendal nerve. These nerves originated from the dorsal rami and penetrated the sacrotuberous ligament. Eisler (1891) identified these nerves as one type of perforating nerves of the sacrotuberous ligament. However, these nerves should be considered medial cluneal nerves with the caudal extension of their origin.

The perforating nerve of the sacrotuberous ligament is often said to be well associated with pudendal nerve entrapment syndrome, which often causes chronic perineal pain (Shafik et al. 1995; Robert et al. 1998; Loukas et al. 2006). Therefore, the ligament is sometimes incised to treat the entrapment (Mauillon et al. 1999; Robert et al. 2005, 2007; Popeney et al. 2007; Filler 2009). The perforating nerve has been reported in recent years by Shafarenko et al. (2022), and their report was similar to that of Eisler (1891). This nerve originates from the pudendal nerve or the root of the pudendal nerve and is distributed in the inferior gluteal region and/or medial upper part of the thigh. Therefore, the nerve should be distinguished from the gluteal branch of the PFC. In the present study, these branches were not found, but if they were found, they would have been found at the transitional zone between the pudendal and tibial nerves, both in origin and course.

### Conclusions

As for the thigh and gluteal branches of the PFC, the dorsoventral order of those originating from the sacral plexus corresponds to the lateromedial order of their distribution. The dorsoventral boundary was displaced from one another at the inferior margin of the gluteus maximus between the thigh and gluteal branches, possibly due to the downward expansion of the gluteus maximus muscle. The perineal branch of the PFC corresponds to the innermost thigh branch and runs through the nerve at the transitional zone between the tibial and pudendal nerves. In addition, the medial part of the inferior gluteal region is distributed by branches originating from the pudendal nerve and/or roots of the pudendal nerve. These branches should be distinguished from the gluteal branches of the PFC. Therefore, the gluteal branches can be classified into the medial and lateral inferior cluneal nerves. Finally, the medial part of the inferior gluteal region is distributed by the branches of the dorsal sacral rami. These branches may represent an expansion of the medial collateral nerve distribution area.

## Data Availability

The datasets used and/or analyzed during the current study are available from the corresponding author upon reasonable request.
